# Supplementary material for: The Abundance of Tumor-Infiltrating CD8+ Tissue Resident Memory T Lymphocytes Correlates with Patient Survival in Glioblastoma
Source: Biomedicines. 2022 Oct 1;10(10):2454. doi: 10.3390/biomedicines10102454 (PMC9599482; doi:10.3390/biomedicines10102454)
Supplement: Supplementary file 1 [file biomedicines-10-02454-s001.zip › biomedicines-1883982-supplementary.pdf]

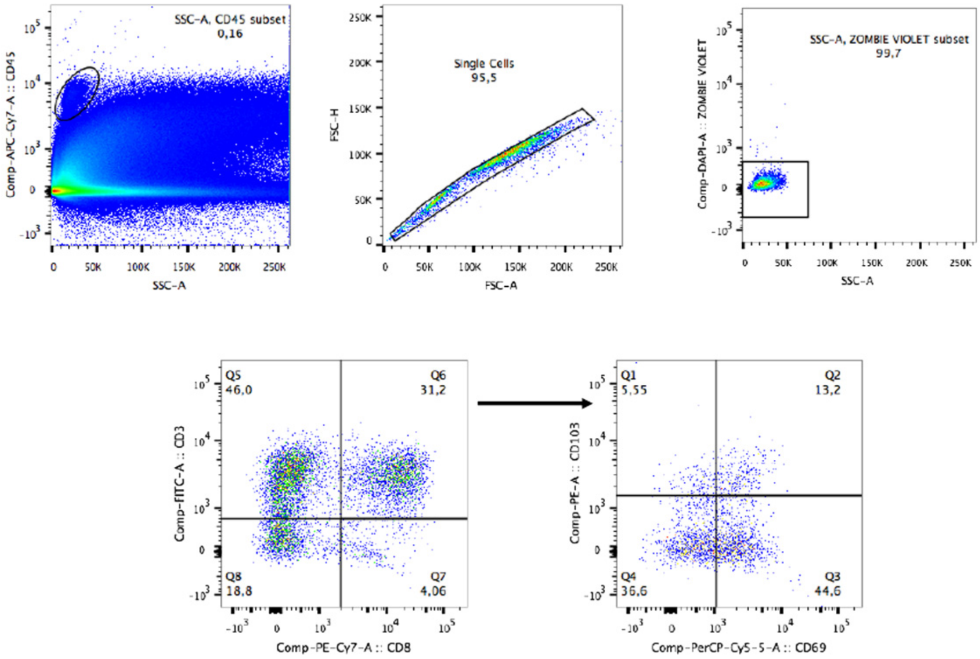

Supplemental Figure S1. Representative dot plots showing the gating strategy used for the identification of CD8+ TRM cells.
